# Trait Emotional Intelligence and Happiness of Young Adults: The Mediating Role of Perfectionism

**DOI:** 10.3390/ijerph182010800

**Published:** 2021-10-14

**Authors:** Siti Khadijah Zainal Badri, Min Yao Kong, Wan Mohd Azam Wan Mohd Yunus, Nor Akmar Nordin, Wai Meng Yap

**Affiliations:** 1 Division of Organisational and Applied Psychology, Faculty of Arts and Social Sciences, University of Nottingham, Semenyih 43500, Malaysia; SitiKhadijah.Zainal@nottingham.edu.my (S.K.Z.B.); kminyao@hotmail.my (M.Y.K.); 2 Department of Psychology, School of Human Resource Development and Psychology, Faculty of Social Sciences and Humanities, Universiti Teknologi Malaysia, Skudai 81310, Malaysia; akmar_nordin@utm.my; 3 Research Centre for Child Psychiatry, University of Turku, 20014 Turku, Finland; 4 INVEST Research Flagship Center, University of Turku, 20014 Turku, Finland; 5 Department of Psychology, HELP University, Shah Alam 40150, Malaysia; waimeng.yap@help.edu.my

**Keywords:** young adult, perfectionism, emotional intelligence, happiness

## Abstract

Perfectionism or a tendency to aim for an unrealistic standard can impair happiness. However, the potential mechanisms of perfectionism to explain the association between trait emotional intelligence (EI) and happiness are still understudied. This study explores the mediating role of perfectionism in the relationship between trait emotional intelligence (EI) and happiness among young adults. A cross-sectional sample of 259 young adults aged between 18 to 35 years old was recruited. All analyses were conducted using SPSS and AMOS Structural Equation Modeling. High trait EI was linked to low perfectionism and high happiness levels. Furthermore, perfectionism mediated the relationship between trait EI and happiness. Although high trait EI lowered maladaptive perfectionism, the negative impact of maladaptive perfectionism remained and subsequently led to decreasing happiness levels of young adults. This study offers an enhanced understanding of the role of perfectionism in explaining the happiness state of young adults. Moreover, it provides practical implications for using trait EI and managing perfectionism tendency to manage the happiness and wellbeing of the young adult population.

## 1. Introduction

Happiness is a positive mental and emotional state ranging from contentment and intense joy [1]. It is commonly discussed under the wellbeing topic and interchangeably used with other terms, including subjective wellbeing. Feeling happy is essential as it leads to meaningful, fulfilling, and satisfying experiences [2]. The notion of happiness propels individual subjective rather than objective evaluation. It depends on various environmental and individual factors such as socioeconomic background, environmental condition or stressors, and cultural expectation [1,3,4,5,6]. The Conceptual Referent Theory of Happiness (CRT) discussed that happiness depends on individual expectation and the underlying concept of being well, which positions happiness as a state that a person overall thinks about their life rather than being solely based on their emotional arousal [7].

The widely accepted definition of emotional intelligence refers to EI as one’s ability to manage the emotional aspect of life and its related events [8]. Meanwhile, trait EI is distinguished as a quality embedded in one’s personality components and part of individual self-perception [9,10]. Arguably, the connection between happiness with personality and emotional intelligence is less established since much research is inclined to emphasize happiness concerning one’s environmental aspect. In this paper, the relationships among perfectionism, trait emotional intelligence (EI), and happiness of the young adult are extended to understand further how perfectionism can serve as a mechanism to explain the link between trait EI and happiness of the young adult. Flett and Hewitt [11] described perfectionism as an act in pursuit of perfection adhered by excessive performance or high standards along with the tendency to overly evaluate and criticize one’s behavior. This act is commonly portrayed in the behavior of comparing or contrasting oneself unrealistically, which results in a lower degree of self-contentment and esteem [12]. Perfectionism or an act to achieve the highest standard [11] is becoming more prevalent among the younger population due to wider social media influence [13]. This act can lead to two circumstances. Firstly, it can help individuals evolve and maximize their potential (refers to adaptive perfectionism). Secondly, it may push an individual into self-destruction under the circumstance of unrealistic perfectionism or refers to maladaptive perfectionism [14].

On the other hand, trait EI relates to one’s self-perception and emotion regulation [9,10]. Its implication is two-sided, with high levels of this trait enabling an individual to acquire more positive accomplishment under good emotional regulation [15], and increasing one’s manipulation ability to achieve a desirable goal [16]. However, there has been a dearth of knowledge about how trait EI may ignite perfectionism, especially among the young adult population [13], and the role of perfectionism to bridge the existing link between trait EI and individual happiness. While the literature continues to highlight the positive influence of EI towards promoting good wellbeing, there has been limited studies that investigated the link among trait EI, perfectionism, and happiness [17]. More importantly, limited knowledge remains on the relationships of perfectionism with trait EI and young adults’ happiness [18], emphasizing the role of perfectionism as a mechanism to explain the connection. Therefore, using a sample of 259 young adults, this study aimed to establish a more precise link between trait EI and young adult happiness. Furthermore, it elucidates the role of one’s perfectionist tendency as a mediator.

### 1.1. EI and Happiness

Mayer et al. [19] posited that individuals with higher trait EI are better at regulating their emotions and, thus, better at managing stresses that are crucial to happiness [20]. Emotional intelligence explains over half of the total variance in happiness [21], and empirical evidence suggests that those higher in trait EI are happier than vice versa [22,23]. Since happiness is related to hopefulness and optimism [24], those with greater trait EI thrive better due to their better competence in managing relationships and events. These circumstances explain why those high in trait EI fare better in satisfaction and happiness [25,26,27] than those with low in trait EI who often encounter difficulty navigating others and their emotions under stressful events [28,29]. Therefore, we present the following hypothesis:

**Hypothesis** **1.** *Higher trait EI is linked to greater happiness among young adults.*

### 1.2. EI and Perfectionism

Gong et al. [30] argued that trait EI was negatively linked to maladaptive perfectionism as those with high trait EI are likely more optimistic when facing failure. A maladaptive perfectionist tendency to misinterpret and over-assess emotional cues from the surrounding has increased the commonness of unhealthy coping mechanisms such as denial among this group of individuals [31]. The desperation to prove self-worth causes those low in trait EI to be more likely to engage in maladaptive perfectionism, which reduces happiness. A previous study also found a positive link between maladaptive perfectionism and depression [32]. Contrastingly, high trait EI is linked to adaptive perfectionism, as hinted by Perrone-McGovern et al. [33]. Due to a low emphasis on evaluation by others, individuals with adaptive perfectionism are more resilient toward negative evaluation [34,35], making them less critical when encountering failure. The good ability of those with high EI to acknowledge and understand internal and surrounding emotional reactions [30] causes them to utilize the perfectionism tendency as a medium to further improve future performance. Therefore, the second and third hypotheses are as follows:

**Hypotheses** **2a** **and** **2b.** *Low trait EI is linked to maladaptive perfectionism and high trait EI is linked to adaptive perfectionism among young adults.*

### 1.3. Perfectionism and Happiness

Perfectionism also influences one’s happiness experience, whereby maladaptive perfectionist has a greater risk of mental distress, negative emotion [36], deteriorated body health [37], and declining wellbeing [38]. Evidence has suggested that incongruence between expectation and outcome predicts lower satisfaction, higher depression, and loneliness among young individuals [39]. The misfit between desired standard and actual performance underlines maladaptive perfectionists’ dissatisfaction and unhappiness due to their decreased flexibility in dealing with incongruence [40]. On the other hand, an adaptive perfectionist is more flexible in adjusting to incongruence and, thus, may acquire greater happiness and satisfaction [41].

**Hypotheses** **3a** **and** **3b.** *Maladaptive perfectionism is linked to low happiness, while adaptive perfectionism is linked to high happiness.*

### 1.4. Perfectionism as a Mechanism Explaining the Relationship between Trait EI and Happiness

**Figure 1:**
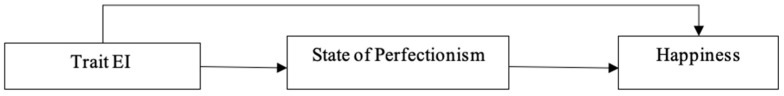
Proposed study framework.

In the past, studies have established direct relationships between (1) EI and perfectionism [29,42,43] and (2) perfectionism and happiness [17], suggesting the potential of perfectionism to act as a mediator in explaining one’s happiness experience. Since perfectionism takes two forms, adaptive and maladaptive, the outcomes of these two conducts are different and may produce a different outcome in bridging the trait EI and happiness. However, little to no study has examined perfectionism as a mediator. The closest framework is related to the role of EI as a moderator between perfectionism and happiness (see Abdollahi et al. [17]). Regardless, evidence states that different perfectionism types predict different levels of happiness outcomes [30]. Notably, according to the Cognitive Theory of Perfectionism (CTP) [44], those who strive for perfection tend to chronically engage in overthinking to fulfill their ‘perfect’ standard and, as such, their intense desire to achieve this standard may cause them to have excessive mistake rumination, failure perseveration, and social comparison. We, thus, further contribute to expanding this view by examining two different types of perfectionism construct, adaptive and maladaptive, as another indicator to further explain the link between trait EI and happiness. We argue that maladaptive perfectionism explains the negative link between trait EI and happiness, while adaptive perfectionism does the opposite. Additionally, our research also builds on the evidence-based of emotional intelligence research, as highlighted by Mayer, Salovey, and Carusso [19], for researchers to investigate the processes underlying EI. Our subsequent hypotheses are outlined below, and the proposed framework is illustrated in Figure 1.

**Hypotheses** **4a** **and** **4b.** *Maladaptive and adaptive perfectionism mediate the relationship between trait EI and happiness.*

## 2. Materials and Methods

### 2.1. Research Design, Sample, and Ethical Considerations

A total of 259 university students were recruited to test our framework. Only those aged 18 to 35 years during the commencement of data collection were eligible to participate in representing the young adult population. Due to time and cost concerns, samples were collected using random sampling via an online platform, Qualtrics. The online survey consisted of three parts: participant consent, demographic, and research instruments sections. A total of 284 samples were retrieved. However, only 259 usable samples were used for hypothesis testing after excluding 20 responses due to incomplete data and five responses due to potential multivariate outliers according to the suggestion from the Mahalanobis distance test. The final samples were deemed adequate on the basis of the recommendations of greater than 200 for SEM research [45] and five or 10 observations per estimated parameters [46].

Before commencing data collection, this study received ethical approval from the Division of Organizational and Applied Psychology, the University of Nottingham. All respondents were informed of their voluntary participation, anonymity terms, and right to withdraw at the point of data collection.

### 2.2. Measures

Happiness was measured using the 29-item Oxford Happiness Inventory (OHQ) scale [47]. Items were rated using a unidimensional six-point Likert scale from 1 “strongly disagree” to 6 “strongly agree”. The scale was chosen for its validity and reliability to evaluate the individual state of happiness, especially among student or adult populations [48,49,50]. A sample item is “I feel like life is very rewarding”. Trait emotional intelligence was measured using the short version of the Trait Emotional Intelligence Questionnaire (TEIQue-SF) by Petrides and Furnham [51]. The scale has been widely tested and relatively stable across different cultural contexts and populations [52,53]. It consists of four subdimensions of wellbeing, self-control, emotionality, and sociability. The scale comprises 30 items rated with a seven-point Likert scale from 1 “completely disagree” to 7 “completely agree”. A sample item from the scale is “I generally believe that things will work out fine in my life”. Perfectionism was measured by the Almost Perfect Scale-Revised (APS-R) by Slaney and Ashby [54]. It consists of 23 items with a seven-point Likert scale from 1 (strongly disagree) to 7 (strongly agree). The score was based on three subdimensions: standards, order, and discrepancy, which were later combined to form adaptive (order) and maladaptive perfection (discrepancy and standard). A sample item is “Neatness is important to me”.

### 2.3. Preliminary Analysis and Method of Data Analysis

Before executing the data analysis, missing data and distribution analysis was performed to observe the overall dataset. Then, validity and reliability tests were carried out on all of the study instruments. Confirmatory factor analysis (CFA) was performed to assess the construct validity of all scales. This method was performed instead of EFA since the factorization of all the instruments has already been well established and widely reported and, thus, does not require exploring underlying factors. The assessment of construct validity was based on model fit indices such as the χ^2^ (df) statistic, Comparative fit index (CFI), Tucker–Lewis index (TLI), incremental fit index (IFI), root-mean-square error of approximation (RMSEA), and the standardized root-mean-square index (SRMR). Values below 0.07 for the SRMR, below 0.08 for the RMSEA, and above 0.95 for the CFI, TLI, and IFI indicate an acceptable model fit [55]. Common method variance (CMV) was assessed using Harman’s single-factor test. Meanwhile, convergent validity was evaluated by looking at average variance extracted (AVE) and composite reliability. For this, the rule of thumb of AVE was applied (higher than 0.50), with reliability above 0.70. However, some exceptions were made in case the AVE was less than 0.5 with composite reliability higher than 0.6, whereby the convergent validity of the construct was still considered adequate [56]. All results were analyzed using Statistical Package for Social Sciences (SPSS) and AMOS Structural Equation Modeling.

## 3. Results

### 3.1. Demographic Profile of the Respondents

In summary, 173 of the respondents were aged between 21 and 30 years old (66.80%), 83 respondents reported being between 18 and 20 years old (32.04%), and three respondents reported being between 31 and 35 years old (1.16%). In terms of study level, the majority of the respondents were undergraduate students with 218 responses (84.20%), followed by foundation students with 26 responses (10.00%) and 15 postgraduate students (5.80%). Additionally, 156 of the respondents were female (60.23%), and 103 were male (30.77%).

### 3.2. Distribution and Common Method Variance

The present data were normally dispersed with skewness values lower than 2 and kurtosis below 7, which aligned with Garson’s statistical suggestion [57]. Common method variance (CMV) was examined using Harman’s single factor using principal axis factoring. The finding suggested no CMV issue as all items loaded as multiple constructs with an average variance percentage of 22.002% [58].

### 3.3. Structural Validity and Reliability of the Instruments

Confirmatory factor analysis (CFA) was performed to assess and confirm construct validity of all scales, with all constructs found to adequately fulfilled the necessary cutoffs. In summary, the Oxford Happiness Questionnaire (OHQ) was found as a single-factor scale (goodness fit index of χ^2^ (259) = 63.17, *p* < 0.001, χ^2^/df = 2.43, RMSEA = 0.07 (90% CI: 0.051–0.098), RMR = 0.050, CFI = 0.960, GFI = 0.950, and TLI = 0.950), yielding a high Cronbach value of 0.91. Moving on, TEIQue-SF was better as a four-factor scale with good fit indices χ^2^ (259) = 280.12, *p* < 0.001, χ^2^/df = 2.46, RMSEA = 0.07 (90% CI: 0.064–0.086), RMR = 0.080, CFI = 0.948, GFI = 0.946, and TLI = 0.945, along with a reliability score of 0.950. Lastly, the perfectionism scale yielded an acceptable fit with χ^2^ (259) = 47.49, *p* < 0.001, χ^2^/df = 1.169, RMSEA = 0.026 (90% CI: 0.000–0.052), RMR = 0.070, CFI = 0.996, GFI = 0.969, and TLI = 0.994, along with a high Cronbach alpha value of 0.891.

### 3.4. Intercorrelation among Variables and Criterion Validity

Table 1 presents the intercorrelation among the studied variables. Both overall and subdimensions (i.e., self-control, sociability, wellbeing, and emotionality) were included for trait EI. Meanwhile, both overall and subdimensions of perfectionism (i.e., maladaptive and adaptive perfectionism) were included for perfectionism. In summary, the result suggested that the emotional intelligence and perfectionism dimensions were correlated from low to moderately high, with happiness suggesting a good criterion validity according to the findings presented in Table 1. On average, student happiness level was at 3.961, which indicated a medium score. Trait emotional intelligence and maladaptive and adaptive perfections scored within the range of 4.261 to 4.900, showing moderate to high levels. Trait emotional intelligence was positively correlated with happiness (0.774), while maladaptive (−0.514) and adaptive perfectionism (−0.503) were negatively linked to both happiness and trait emotional intelligence (maladaptive perfectionism = −0.513; adaptive perfectionism = −0.535).

### 3.5. Hypothesis Testing

**Figure 2:**
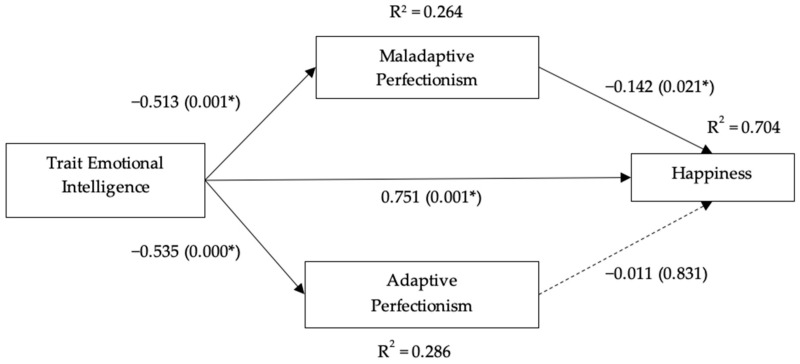
Summary of the path analysis findings (* for significant *p*-values).

Mediation analysis was conducted using the path analysis performed using AMOS version 26 of 5000 bootstrapping samples with 95% bias correction (BC). The academic level variable was controlled since EI score may increase from young adult to middle age [59]. Table 2 and Figure 2 summarize the findings. Trait EI and perfectionism contributed 70.4% variance in students’ happiness. The overall path model showed a good fit with χ^2^ (259) = 0.299, *p* < 0.001, χ^2^/df = 0.100 RMSEA = 0.00, RMR = 0.003, CFI = 1.00, GFI = 1.00, and TLI = 0.99. Specifically, trait EI was linked negatively to both maladaptive (β = −0.513) and adaptive perfectionism (β = −0.535), while it was linked positively to happiness (β = 0.751); thus, hypotheses 1 and 2a are supported, while hypothesis 2b is rejected. Moving on, only maladaptive perfectionism (β = −0.142) was negatively correlated with students’ happiness, but adaptive perfectionism was not (β = −0.011); therefore, only hypothesis 3a is supported. Lastly, maladaptive perfectionism was found as a significant mediator between trait EI and happiness. That is, high trait EI was linked to low maladaptive perfectionism, which in turn lowered happiness levels (indirect effects = 0.059, *p* < 0.001, BC (LLCI = 0.011, ULCI = 0.109). Accordingly, only hypothesis 4a was supported.

## 4. Discussion

In this paper, happiness was examined focusing on young adults’ positive emotional state and how it can be influenced by emotional intelligence (EI) and perfectionism. The motivation of this study to pursue this line of research is in tying together these three different subjects and the social reality faced by young adults, who often struggle to keep up with their emotional experience, as well as the lack of studies done among this population. This population was chosen due to two main reasons. First, there is a rising concern over young adults’ happiness due to immense social, academic, social, and peer pressure, increasing mental health and suicide ideation risk [60]. Second, this cohort has an increasing perfectionist tendency to conform to social expectations resulting from heavy social media usage [11,61].

Studies have proven that happiness is linked to self-esteem, motivation, and performance [62,63,64,65,66,67,68]. This is partly due to the association between a happy state with greater motivation and positive willpower toward embracing different life events [69]. Although antecedents and outcomes of happiness have been well recognized, the evolved social landscape and heightened cultural expectation have led to a requestioning of the applicability of past studies to accurately represent the current population. More importantly, studies on happiness underline the significance of examining happiness using a smaller unit and not generalizing the happiness experienced as a comprehensive model [70].

This study suggests that a high state of emotional intelligence was linked to increasing young adults’ happiness levels. Previous studies have suggested that happiness is parallel to one’s ability to control and manage emotions [71]. The ability to regulate one’s emotion, especially in filtering how individuals think and perceive their surroundings, is essential to fostering happiness in the young adult generation [70]. Many empirical studies have mentioned the distinction in the quality of today’s generation of young adults having lower emotional aptitude due to a lack of understanding and control toward their emotional discontent [72]. Branded as a ‘sheltered’ generation in many articles, this particular cohort is used to being protected from failure by their guardians. This has led to this particular generation experiencing more significant distress once they experience failure, which has led to them experiencing emotional discontent. In this study, it was found that high emotional intelligence increased happiness. Such a result ascertains the importance of refining EI ability among the young adult population. It indicates the vulnerability of those with lower EI to experience lower happiness levels, which needs further attention.

It was also discovered that EI is associated with the state of perfectionism, which in turn determines young adults’ happiness. The notion of perfectionism believes in the dual effects of this construct. Positive perfectionism, commonly known as adaptive perfectionism, is associated with positive antecedents and outcomes. Meanwhile, the reverse type, recurrently mentioned as maladaptive perfectionism, is linked to negative antecedents and outcomes. Although this is true for some populations, different evidence was found in this study. Although the possession of high EI replicates more positive outcomes, it is partly true when it is linked to perfectionism. Instead, under high EI, young adults’ adaptive perfectionism becomes lower, although many studies have declared the popularity of this construct to improve the quality of life and performance. This advocates that higher EI reduces the likeliness for overall perfectionism behavior to be projected regardless of its occurrence in negative or positive forms.

Interestingly, this study established significant evidence on the mechanism of perfectionism in bridging EI with happiness. Specifically, high EI lowers maladaptive perfectionism and, thus, decreases happiness. This evidence was found to be intriguing in several ways. First, although high EI lowers maladaptive perfectionism, the negative result of being perfectionist remained and subsequently led to decreasing happiness levels. Such a finding indicates that the condition of high EI does not transfer enough positive influence to diminish the negative effect of engaging in perfectionist behavior, thus reducing young adult happiness. Second, the mechanism only existed under the portrayal of maladaptive perfectionism, but not adaptive perfectionism. This suggests the limited ability of EI to carry the influence of perfectionism behavior, which subsequently influences young adults’ happiness. Third, this study’s overall portrayal of how EI, perfectionism, and happiness are linked indicates the detrimental influence of engaging in perfectionist behavior on wellbeing. Regardless of whether the behavior is performed positively or negatively, the outcome is nonetheless unfavorable to one’s happiness experience.

## 5. Conclusions

This study contributes to the current literature in several ways. First, it adds evidence on the role of trait EI toward increasing young adult happiness. Such a result signals the need to further focus on building the EI of the young adult population for their happiness. Some studies have reported that the younger generation has a lower level of competence in managing their emotion [73]. While many factors can contribute to one state of EI, such as parenting [74] and exposure to social media [75], exploring relevant factors is crucial. This is particularly relevant to find ways and proper intervention in helping them to further develop this aspect. Second, this study extends the current understanding by explaining how young adults’ state of perfectionism may explain their decreasing happiness. Notably, the findings where high EI was found to reduce perfectionism level regardless of its type have proven the importance of nurturing and developing good emotional endurance among the young adult population in helping this cohort achieve good wellbeing conditions aligned with the past evidence [76]. Furthermore, this study also shows that, regardless of perfectionism types, whether constructive or destructive, it only reduces or does not produce any influence on young adults’ happiness. Evidence has suggested that positive perfectionism or adaptive perfectionism can be a push factor to improve one’s performance and motivation, as well as reduce academic procrastination [77], but this is not the case for wellbeing and happiness. Thus, the portrayal of this behavior should be carefully monitored as it can act as a double-edged sword towards individuals’ subjective experiences. What is more important is that such behavior can make individuals less flexible when facing challenging life events, thus becoming more prone to psychological distress.

### 5.1. Implications

Higher-education institutions with young adults as their major population can develop an emotional intelligence profiling for their students. They can use the profiling of EI competency to develop a specific curriculum for students. Higher-education institutions could also develop emotional intelligence workshops to address the needs of this cohort. Currently, most emotional intelligence workshops have been implemented for teachers and middle-school students [78], while the focus on young adults remains lacking. Furthermore, with most academic settings being focused on EI and academic performance, universities should focus on conversations concerning happiness, considering that young adults are at increased risk of mental health issues, especially during this pandemic [79]. Awareness should also be instilled among young adults of the possible negative consequences of being perfectionistic (whether maladaptive or adaptive), and how it might influence their happiness levels.

Perfectionism, as shown in this study, can lead to unhappiness, which in the long run might contribute to burnout and decreased mental health. Another applied setting that can be explored is organizations employing young adults, by developing intervention programs and workshops surrounding emotional intelligence and perfectionism. Specifically, the conversation on how perfectionism, whether positive or negative, can decrease one’s happiness levels is essential, considering the increasingly fast-paced and high expectations of the working world. Moreover, young adults are increasingly falling into the trap of feeling pressured to succeed [80], which might validate the need for their perfectionistic tendencies as a survival mechanism. Conversations surrounding this are essential, with evidence demonstrating that unhappy employees are more likely to exhibit adverse workplace outcomes such as lower job satisfaction [81].

### 5.2. Limitations

Similar to other studies, this study was not without any limitations. As it focused only on young adults, the result can only be generalized toward those who share similar characteristics with this population. As generation plays a huge role in shaping individual behavior, future research might expand this study to other populations or cultures. Worth investigating is whether there are any differences in the outcome when replicating the study in a different population. Second, although the likelihood of parenting and social media to act as a catalyst to perfectionism was mentioned several times, this was not examined in the current paper. Therefore, it would be interesting if a future study considers those factors so that a more comprehensive model can be developed, leading to more centralized suggestions and discussions for the future. Additionally, this study employed a cross-sectional quantitative design, which did not allow causal inference. Future studies, therefore, can replicate this study and expand it using other approaches such as mixed-methods or longitudinal designs to derive a better conclusion. Furthermore, examining the issue using qualitative analyses would be interesting to allow more in-depth and richer information regarding this situation.

## Figures and Tables

**Table 1:** Demographic and intercorrelation between variables.

Variables	Range	Mean	SD	H	TEI	SC	Soc	WB	Emo	Per	Mal	Ada
Happiness	1–6	3.961	0.887	1	0.774 **	0.605 **	0.532 **	0.833 **	0.299 **	−0.534 **	−0.514 **	−0.503 **
Overall Trait EI	1–7	4.635	0.792	0.774 **	1	0.784 **	0.799 **	0.862 **	0.584 **	−0.540 **	−0.513 **	−0.535 **
Self-Control	1–7	4.590	1.022	0.605 **	0.784 **	1	0.515 **	0.695 **	0.248 **	−0.471 **	−0.458 **	−0.429 **
Sociability	1–7	4.261	0.976	0.532 **	0.799 **	0.515 **	1	0.514 **	0.358 **	−0.390 **	−0.352 **	−0.454 **
Wellbeing	1–7	4.900	0.980	0.833 **	0.862 **	0.695 **	0.514 **	1	0.304 **	−0.519 **	−0.499 **	−0.493 **
Emotionality	1–7	4.772	1.192	0.299 **	0.584 **	0.248 **	0.358 **	0.304 **	1	−0.244 **	−0.239 **	−0.216 **
Perfectionism	1–7	4.444	1.149	−0.534 **	−0.540 **	−0.471 **	−0.390 **	−0.519 **	−0.244 **	1	0.990 **	0.848 **
Maladaptive Perfectionism	1–7	4.426	1.152	−0.514 **	−0.513 **	−0.458 **	−0.352 **	−0.499 **	−0.239 **	0.990 **	1	0.763 **
Adaptive Perfectionism	1–7	4.523	1.405	−0.503 **	−0.535 **	−0.429 **	−0.454 **	−0.493 **	−0.216 **	0.848 **	0.763 **	1

** *p* < 0.005. H—happiness, TEI—overall trait emotional intelligence, SC—self-control, Soc—sociability, WB—wellbeing, Emo—Emotionality, Per—perfectionism, Mal—maladaptiveness, Ada—adaptiveness.

**Table 2:** Regression results and mediating effects of perfectionism.

	β	SE	Sig	LLCI	ULCI
**Direct effects paths**					
Trait EI → happiness	0.751 ***	0.033	0.001	0.681	0.810
Trait EI → maladaptive perfectionism	−0.513 ***	0.050	0.001	−0.605	−0.409
Trait EI → adaptive perfectionism	−0.535 ***	0.048	0.000	−0.619	−0.403
Maladaptive perfectionism → happiness	−0.142 **	0.061	0.020	−0.262	−0.021
Adaptive perfectionism → happiness	−0.011	0.060	0.831	−0.132	0.105
Academic level → trait EI (controlled)	0.070	0.065	0.276	−0.054	0.201
**Indirect effects from trait EI to happiness**					
Total indirect effect (path a + b)	0.064 ***	0.028	0.001	0.028	0.105
via maladaptive perfectionism (path a)	0.059 ***	0.035	0.016	0.011	0.109
via adaptive perfectionism (path b)	0.005	0.042	0.831	−0.44	0.831

Significant at *** *p* < 0.001, ** *p* < 0.005.

## Data Availability

The data presented in this study are available on request from the first author.
